# Biophysical Modulation of the Mitochondrial Metabolism and Redox in Bone Homeostasis and Osteoporosis: How Biophysics Converts into Bioenergetics

**DOI:** 10.3390/antiox10091394

**Published:** 2021-08-30

**Authors:** Feng-Sheng Wang, Re-Wen Wu, Yu-Shan Chen, Jih-Yang Ko, Holger Jahr, Wei-Shiung Lian

**Affiliations:** 1 Core Laboratory for Phenomics and Diagnostic, Department of Medical Research and Chang Gung University College of Medicine, Kaohsiung Chang Gung Memorial Hospital, Kaohsiung 83301, Taiwan; wangfs@ms33.hinet.net (F.-S.W.); ggyy58720240@gmail.com (Y.-S.C.); 2 Center for Mitochondrial Research and Medicine, Kaohsiung Chang Gung Memorial Hospital, Kaohsiung 83301, Taiwan; 3 Department of Orthopedic Surgery, Chang Gung University College of Medicine, Kaohsiung Chang Gung Memorial Hospital, Kaohsiung 83301, Taiwan; ray4595@cgmh.org.tw (R.-W.W.); kojy@cgmh.org.tw (J.-Y.K.); 4 Department of Anatomy and Cell Biology, University Hospital RWTH, 52074 Aachen, Germany; h.jahr@masstrichtuniversity.nl; 5 Department of Orthopedic Surgery, Maastricht University Medical Center, 6229 ER Maastricht, The Netherlands

**Keywords:** osteoblasts, osteoporosis, biophysical stimulation, mechanosensitive, mitochondria biogenesis, PGC-1α, Fndc5, Irisin, Sirt3, Nrf2

## Abstract

Bone-forming cells build mineralized microstructure and couple with bone-resorbing cells, harmonizing bone mineral acquisition, and remodeling to maintain bone mass homeostasis. Mitochondrial glycolysis and oxidative phosphorylation pathways together with ROS generation meet the energy requirement for bone-forming cell growth and differentiation, respectively. Moderate mechanical stimulations, such as weight loading, physical activity, ultrasound, vibration, and electromagnetic field stimulation, etc., are advantageous to bone-forming cell activity, promoting bone anabolism to compromise osteoporosis development. A plethora of molecules, including ion channels, integrins, focal adhesion kinases, and myokines, are mechanosensitive and transduce mechanical stimuli into intercellular signaling, regulating growth, mineralized extracellular matrix biosynthesis, and resorption. Mechanical stimulation changes mitochondrial respiration, biogenesis, dynamics, calcium influx, and redox, whereas mechanical disuse induces mitochondrial dysfunction and oxidative stress, which aggravates bone-forming cell apoptosis, senescence, and dysfunction. The control of the mitochondrial biogenesis activator PGC-1α by NAD+-dependent deacetylase sirtuins or myokine FNDC/irisin or repression of oxidative stress by mitochondrial antioxidant Nrf2 modulates the biophysical stimulation for the promotion of bone integrity. This review sheds light onto the roles of mechanosensitive signaling, mitochondrial dynamics, and antioxidants in mediating the anabolic effects of biophysical stimulation to bone tissue and highlights the remedial potential of mitochondrial biogenesis regulators for osteoporosis.

## 1. Introduction

Osteoporosis is characterized by very poor bone quality, including loss in the bone mineral density, microarchitecture, and mechanical strength, etc., and is the leading etiological cause of skeletal fragility and dysfunction in postmenopausal women and elderly people [1]. Bone-making cells synthesize the bone matrix to form a finely woven mineralized microarchitecture. These cells also produce cytokines or couple with bone-resorbing cells, dynamically keeping the bone turnover unfluctuating [2]. 

Bone-forming cells consume a considerable amount of energy to maintain bone formation capacity and balance the remodeling activity. The mitochondrial metabolism is an indispensable process in producing the cellular energy component adenosine triphosphate (ATP), which powers cells for survival and to remain functional. Mitochondrial dysfunction induces reactive oxygen species (ROS) overproduction, aggravating oxidative stress to cellular microenvironment [3]. Promoting the mineral acquisition capacity of bone-making cells by bone anabolic factors, including vitamin D, the Wnt pathway, bone morphogenetic protein, and parathyroid hormone (PTH), etc. facilitates the net gain of bone mass, keeping skeletal tissue strong [4,5]. 

The Food and Drug Administration (FDA)-approved anti-osteoporosis agents, Romosozumab and Denosumab are found to neutralize sclerostin (a Wnt pathway inhibitor) and osteoclastogenic cytokine RANKL respectively, reducing the risk of osteoporotic fracture. Teriparatide, a recombinant PTH (1–34), promotes osteoblastic activity and bone formation to delay menopause or glucocorticoid excess-mediated osteoporosis [6]. We deliver new insights into the biological function of mitochondrial bioenergetics and redox to bone-forming cells for bone tissue integrity. 

Regular exercise, moderate weight loading, and physical activities, in concert with skeletal muscle, are advantageous to bone health [7]. However, unloading, microgravity, and mechanical disuse are found to dysregulate bone formation and remodeling reactions, accelerating bone mass loss and osteoporosis development [8]. The mechanosensitive nature of skeletal tissue prompts the development of various types of biophysical stimulation interventions, which provide axial mechanical or oscillatory force or pulsed stimuli to enhance bone formation in osteoporotic or fractured bones [9]. 

Bone-making cells adapt to extracellular biophysical stimuli through modulating intracellular signaling transductions to change skeletal metabolism and microenvironment homeostasis [10]. A plethora of molecules, including PIEZOs, TRPVs, integrins, cytoskeletons, and myokines “sense” biophysical stimuli, activating downstream signaling cascades to regulate growth and differentiation of bone-forming cells, bone regeneration, and osteoporosis development [11]. The contribution of mechanotransduction to mitochondrial activity and redox in bone-forming cells and to the bone microenvironment warrants a wide scope of review. 

This review illustrates the role of the mitochondrial metabolism in bone-forming cell function and bone anabolism, the effect of biophysical stimulation on mitochondrial activity and mechanosensitive pathways in bone-forming cells, and the remedial potential of mechanosensitive molecules and mitochondrial metabolism regulators for osteoporosis. 

## 2. The Role of Mitochondrial Homeostasis in Bone-Forming Cell Function

**Figure 1:**
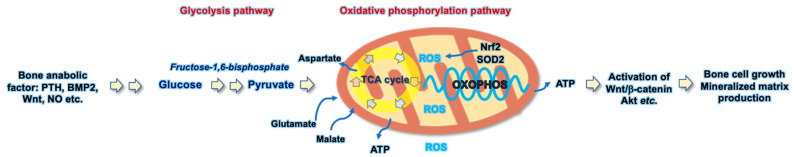
Schematic diagram showing the glycolysis and oxidative phosphorylation pathways involved in the bone-forming cell anabolism.

Mitochondria are powerhouses in the cellular microenvironment, metabolizing glucose, amino acids, and fatty acids and producing ATP and ROS through well-organized tricarboxylic acid (TCA) cycle and electron transfer chains. Mitochondrial bioenergetics is indispensable in sustaining the growth, differentiation, and biological function of bone-forming cells in response to anabolic factors, including bone morphogenetic proteins (BMPs), PTH, Wnt, and nitric oxide (NO) [3] (Figure 1). 

This organelle also provides energy to protect cells from extracellular stresses. Mitochondrial dysfunction induces metabolism repression, electron transfer chain disruption, and redox imbalance, accelerating cellular senescence or apoptotic programs [3]. On the other hand, the disposal of unwanted mitochondrial macromolecules through a sophisticated autophagy program, which is referred as mitophagy, maintains intercellular microenvironment integrity. This dynamic process is required to maintain cellular homeostasis, whereas mitophagy defects induce oxidative stress, thus, accelerating cell dysfunction.

### 2.1. Mitochondrial Bioenergetics and Metabolism 

Mitochondrial ATP is biosynthesized through glycolysis or oxidative phosphorylation pathways [12]. The mitochondrial energetics pathways involve bone-forming cell growth and differentiation. The inhibition of oxidative phosphorylation by antimycin A, an inhibitor for cellular oxidative phosphorylation, impairs osteogenic gene expression and extracellular matrix synthesis in C3H10T1/2 mesenchymal progenitor cells. Forced oxidative phosphorylation by replacing glucose with galactose promotes osteogenesis. This effect activates β-catenin, a master Wnt signaling component, through an acetyl-CoA-dependent acetylation reaction [13]. 

Oxidative phosphorylation is a prominent reaction of ATP generation in nondifferentiated MC3T3-E1 osteoblasts, whereas glycolysis dominants the bioenergetic activity in differentiated osteoblasts [14]. ATP in mature osteoblasts is largely produced through the malate metabolism in the aerobic glycolysis pathway [15]. In addition, different extracellular stimuli or stresses result in different mitochondrial metabolism or metabolite profiles of bone-forming cells. Mitochondrial metabolomic profiles of osteogenesis in human umbilical cord mesenchymal stem cells depend on bone-anabolic agents BMP-2 and glucocorticoid in media [16]. 

Wnt3a and BMP2 drive the mitochondrial metabolism program toward oxidative phosphorylation and activate Akt signaling, which promotes the differentiation of osteoblasts in murine long bone and calvaria bone [17]. The inhibition of nitric oxide production by interrupting argininosuccinate lyase represses aerobic glycolysis and osteoblastic activity. Mice deficient in argininosuccinate lyase develops low bone mass and less osteogenic differentiation capacity than wild type mice [18]. Loss of the leucine-rich repeat-containing G protein coupled receptor 4 (Lgr4) increases aerobic glycolysis and induces osteogenesis loss of osteoprogenitor cells. Lgr4 knockout mice show phenotypes of poor bone microstructure and decreased ex vivo osteogenic differentiation [19].

Mouse embryonic fibroblasts lacking β-actin exhibit high osteogenic differentiation capacity and hydroxyapatite microcrystal overproduction together with increased oxidative phosphorylation programs [20]. The deletion of peroxisome proliferator-activated receptor-δ reduces mitochondrial respiration and shifts the mitochondrial metabolism toward glycolysis, as well as inhibits osteoblast differentiation and bone mass [21]. 

Likewise, the enhancement of oxidative phosphorylation by lactate dehydrogenase inhibitor oxamate increases mineralized matrix production and attenuates estrogen deficiency-induced bone loss. [22]. Manipulation of mitochondrial oxidative phosphorylation programs using pharmaceuticals appears to be a new biomedical strategy to control bone mineral acquisition or steer the osteogenic lineage specification of mesenchymal stem cells for skeletal tissue bioengineering. 

### 2.2. Mitochondrial ROS and Antioxidants

Increased ROS production together with oxidative phosphorylation are present during osteocyte differentiation. Mitochondrial ROS homeostasis is balanced by mitochondrial antioxidants, including nuclear factor E2 p45-related factor 2 (Nrf2). Mice lacking Nrf2 specifically in osteocytes and osteoblasts show phenotypes of low bone mass and repressed trabecular morphology. Administration with Nrf2 agonist dimethyl fumarate compromises osteoporosis development in ovariectomized mice [23].

Growth hormone receptor deletion induces mitochondrial dysfunction, including membrane potential loss, ATP underproduction, and respiration repression rather than glycolysis in osteocytes isolated from male mice. Growth hormone signaling loss also aggravates ROS burst and antioxidant glutathione loss. Mice deficient in growth hormone receptor develop low trabecular bone volume together with decreased biomechanical strength [24]. 

An iron overload activates a permeability transition pore in mitochondria, inducing ROS overproduction and necroptosis in osteoblasts. Antioxidant N-acetylcysteine reverses ROS-mediated osteoblast ferroptosis [25]. Advanced oxidation protein products upregulate NADPH oxidase-mediated ROS generation, impairing mitochondrial function and the survival of osteoblasts. These oxidative products are found to repress bone mineral density and trabecular microstructure of tibiae and vertebrae in aged rats [26]. Chlorogenic acid promotes the Nrf2/HO-1 pathway, which compromises glucocorticoid excess-induced mitochondrial superoxide (O_2_^-^) overproduction, senescence, and apoptosis in murine osteoblasts [27]. 

### 2.3. Mitophagy

Mitophagy involves the clearance of unwanted or dysfunctional mitochondrial organelles to maintain cellular homeostasis through PTEN-induced putative kinase 1 (Pink1) and Parkin-mediated autophagosome formation and ubiquitination. Mitophagy dysfunction is found to induce a plethora of biological activities deleterious to tissue integrity and function [28]. The deletion of mitochondrial deacetylase Sirt3 inhibits oxidative phosphorylation, inducing ROS overproduction and autophagy loss in osteoclasts. Sirt3 deletion accelerates age-induced osteoporotic bone development together with impaired osteoclastic resorption. Pharmacological inhibition of Sirt3 by LC-0296 reverses Sirt3 loss-induced mitochondrial dysfunction and estrogen deficiency-mediated excessive bone resorption and bone mass loss [29].

Sirt3 signaling loss also impairs mitochondrial function and mitophagy, accelerating advanced glycation end products-induced senescence in mesenchymal stem cell and age-mediated osteoporotic bone development. Forced Sirt3 expression preserves mitophagy and osteoporosis in SAMP6 mice, an in vivo model showing accelerated senescence [30]. The promotion of autophagy by mTOR/PI3K signaling inhibitor rapamycin increases the osteogenic activity of bone-marrow mesenchymal stem cells, whereas mitophagy repression by hydroxychloroquine accelerates the senescence program [31]. 

### 2.4. Mitochondrial DNA Mutation and Osteoporosis

Increasing clinical evidence has shown that mitochondrial DNA mutation correlates with low bone mass, especially in the patients with inherited metabolic diseases. For example, a case-control study reveals reductions in the bone mass, cortical thickness, and estimated biomechanics of lumbar spine, hip, and femoral head in patients with the m.3243A>G mutation [32]. Patients with maternally inherited diabetes and deafness carry mitochondrial m.3243A>G mutation and have osteoporosis signs [33]. 

Mitochondrial dysfunction together with a loss in osteogenic differentiation potential are present in mesenchymal stem cells isolated from urine of patients carrying m.3243A>G mutation [34]. The single nucleotide polymorphism of aldehyde dehydrogenase 2 (ALDH2) at rs671 loci correlates with osteoporosis development and osteoporotic hip fracture [35]. Mice lacking the Aldh2 gene show high bone mass phenotypes and increased responses to the bone anabolic actions of PTH treatment [36]. 

## 3. Biophysical Stimulation Intervention for Promoting Bone Integrity

Moderate weight loading, regular exercise, or workouts together with nutritious diets with abundant calcium and vitamin D are advantageous to maintain bone mass, reducing the risk of osteoporotic fracture [7]. Bone tissue adapts to biophysical stimulation, depending on the type, magnitude, and duration of stimuli, by altering bone formation or remodeling reaction. A plethora of physical or electromechanical interventions are found to slow bone mass loss in human osteoporosis and in experimental osteoporosis models. We shed light onto the response of osteoporotic bone tissue in human and in laboratory animals upon interventions as shown in Table 1.

### 3.1. Exercise or Physical Activity

The beneficial effects of physical activity or regular exercise are sporadic. Children or adolescents have better mineral content and density in vertebral, limb, arm, and skeletal muscle development upon regular sporting or physical activities, including jumping, running, gymnastics, and impact sports [37]. Randomized controlled trials reveals that regular exercises enable senior participants to have better mobility, gait speed, and 6-min walking distance than usual care participants [38]. 

The lumbar spine bone mass, stature, muscle strength, and fracture risk of postmenopausal women are improved upon 40-min high-intensity resistance and impact training (twice a week) for a consecutive 8 months [39]. Postmenopausal women upon team handball practices show better bone mineral density and content of the lumbar spine and postural balance than the control participants. Serum bone formation and resorption markers, including osteocalcin and C-telopeptide of type I collagen, are improved upon this practice [40]. Dynamic resistance exercises together with whey protein, vitamin D, and calcium supplementation improve skeletal muscle mass and strength rather than the hip bone mineral density of participants with osteopenia or sarcopenia [41]. 

The loss in the trabecular area, bone mineral content, and bone strength of the distal radius is compromised in middle age and older males upon 8-month high-intensity resistance and impact training [42]. While increasing studies reveal the positive effects of exercises or physical activities to bone quality, a systemic database study of Gibbs et al. suggests that further clinical trials with randomized controlled designs and bigger sample sizes are essential to evaluate the influence of physical activity interventions on vertebral fracture risk or pain in postmenopausal women or in the elderly [43]. 

### 3.2. Electromechanical Stimulation Intervention

The mechano-responsive nature of skeletal tissue to repetitive physical activities rationalizes the utilization of electromechanical interventions for bone formation or osteoporosis prevention. Various types of electromechanical interventions, including mechanical loading, vibration, pulsed electromagnetic fields, and low-intensity pulsed ultrasound, etc., produce high (low) frequencies of pulsed waves through piezoelectric transducer or repetitive weight and mechanical loading., which have been used for in vitro or in vivo models of bone-forming cell dysfunction or osteoporosis. 

#### 3.2.1. Vibration Intervention

A randomized placebo-controlled trial reveals that postmenopausal women with pre-osteoporosis signs have better trabecular volumetric bone mineral density, tibiae stiffness, and marrow fat volume upon 10 min/day, 0.3 g, and 30 Hz whole-body vibration for 1 year than the placebo group [44]. A randomized controlled trial shows that the bone mineral density of femoral neck and lumbar spine in postmenopausal women upon 6-month whole- body vibration are more than in the control group. The serum osteocalcin levels rather than C-telopeptide of type I collagen levels are decreased in the whole-body vibration intervention group [45]. 

The bone mineral density of the hip, lumbar spine, and femoral neck are increased in artistic swimmers upon whole-body vibration [46]. Postmenopausal women have more vertebral bone mineral density in lumbar regions upon a combined intervention with whole-body vibration and parathyroid hormone medication for 12 months as compared to medication only [47]. In experimental osteoporosis models, 0.3 g, 90 Hz low magnitude vibration intervention for 12 weeks improves bone mineral density and strength together with increased osteogenic activities of bone-marrow mesenchymal cells in aged rats [48]. Low level whole-body vibration also attenuates the loss in bone mineral density, cancellous and cortical microstructure, and strength of alloxan-induced diabetic rabbits [49]. 

Whole-body vibration intervention has positive effects on bone mass in young rats over that in aged animals, where the bone mineral density is improved upon a combined intervention with whole-body vibration and a pulsed electromagnetic field [50]. While certain randomized clinical trials have shown appreciating improvement of the bone quality in postmenopausal women, clinical trials with multiple centers or bigger sample sizes warrant further investigations. The biological responses of osteoporotic skeletal tissue to vibration intervention in experimental osteoporosis models appear to depend on the animals’ age, gender, or the magnitude of whole-body vibration. 

#### 3.2.2. Mechanical Loading

Low and high strain of voluntary upper extremity compressive loading enables healthy women around 21–40 years old to have better bone mineral density and content of the ultradistal radius [51]. Upon high-impact mechanical loading with one-legged jumps for 3 months, postmenopausal women’s bone material strength index rather than bone mineral density or microarchitecture is more than the control legs [52]. In experimental animals, knee loading significantly upregulates the tibiae bone mineral density and trabecular structure in ovariectomized mice [53]. 

Axial loading onto the tibiae promotes the bone mineral density of the proximal region of tibiae in young and aged mice. This intervention also attenuates sciatic neurectomy-induced cortical bone loss [54]. In addition, spinal loading slows the loss in bone mineral density and the trabecular area together with increased osteoblast differentiation and inhibited osteoclast formation in ovariectomized mice [55].

#### 3.2.3. Pulsed Electromagnetic Fields and Low-Intensity Ultrasound

Pulsed electromagnetic fields intervention for 4 weeks compromises the loss in trabecular microstructure and morphology together with increased osteogenic gene expression and reduced osteoclastogenic gene expression in bone tissue of ovariectomized mice [56]. The protection effects of 30 T/s pulsed electromagnetic field intervention on cortical bone microstructure and resorption in ovariectomized rats are comparable to the anti-resorptive effect of alendronate medication [57]. 

This intervention also reverses the loss in trabecular and cortical bone integrity, bone strength, osteoblast surface, bone formation rate, and serum osteocalcin levels in rats with spinal cord injury [58]. While low-intensity pulsed ultrasound intervention is found to accelerate fracture healing and attenuate hindlimb suspension-induced bone mass loss in rats [59], little is known about whether these interventions affect menopause or age-induced osteoporosis.

### 3.3. Unloading, Microgravity, and Disuse 

Bone tissue in microgravity conditions develops low bone mass together with decreased mechanical strength. For example, astronauts lose bone mineral density, trabecular volume, and strength upon spaceflight for 6 months together with increased serum bone turnover makers. Resistive exercise attenuates spaceflight-induced loss in bone mass and trabecular thickness [60]. A similar study conducted by Sibonga et al. also show that long-term spaceflight accelerates bone loss in astronauts [61]. 

In hindlimb suspended rodents as an in vivo model of microgravity or disuse, this manipulation accelerates the loss in bone mass, structure, and strength in rats [62]. Mechanical strain interventions compromise hindlimb suspension-induced loss in bone mass and bone geometry, as well as increases serum bone formation markers [63]. These findings explain the mechanosensitive nature of bone tissue and the anabolic effect of biophysical stimulation intervention to bone tissue integrity. 

### 3.4. Limitation of Biophysical Stimulation for Osteoporosis

Kinematic movement or physical activity are well coordinated by skeletal muscle and bone tissue, whereas limb unloading, or disuse is found to impair muscle strength. The possibility cannot be ruled out that physical exercises or biophysical stimulations also influence skeletal muscle function, which may change bone mass, quality, and biomechanical strength. These biophysical interventions may directly or indirectly impact to myoblast function and skeletal tissue microenvironment. Having productive insights into the cellular and molecular interplay of skeletal muscle and bone tissue microenvironment may explain the positive effects of biophysical stimulation on skeletal tissue anabolism and microstructure integrity.

The limitations of electromechanical interventions by pulsed electromagnetic fields, ultrasound, and mechanical loading for slowing down osteoporotic skeletal tissue development that should be acknowledged are that poor bone quality is ubiquitously present in menopause or age-induced osteoporotic diseases. Little is known about whether local stimulation can induce ubiquitous effects on all skeletal tissues and whether these proof-of-concept investigations of experimental osteoporosis models can be extrapolated to human osteoporosis. Understanding about the molecular mechanisms by which biophysical stimulation promotes bone cell activity, bone formation, and osteoclastic resorption activity facilitates the development of remedial strategies for osteoporotic disorders. 

## 4. Mechanosensitive Molecular Mechanism Underly Bone Mass Homeostasis 

Expanding analysis shows that signaling transductions or gene transcription in bone-forming cells is changed upon biophysical stimulation. These molecules regulate downstream signaling cascades, modulating survival and anabolic activities. Mechanosensitive molecules play an important role in converting extracellular mechanical stimuli into biological signals. For example, mechanical stress alters cytoskeleton organization and contraction, prompting integrin signaling to activate TGF-β1 in chondrocytes [64]. 

Emerging evidence from gain (loss) of function studies has uncovered that cytoskeleton, integrin, myokine, and ion channels, etc., appear to “sense” mechanical stimulation or physical activity on skeletal tissue. Bioinformatics searches on Cytoscape (http://cytoscaple.org, accessed on 27 July 2021) and Genecards (www.genecards.org, accessed on 27 July 2021) reveal the putative interactions of mechanoresponsive molecules and intracellular signaling transductions, which involve a plethora of biological activities (Table 2). The generation of mice deficient in mechanosensitive molecules specifically in osteoprogenitors, osteoblasts, osteocytes, or osteoclasts do prompt us to have an insight into the role of these molecules in mechanical stimulation or disuse-mediated skeletal tissue anabolism and deterioration. 

### 4.1. Ion Channels

Of ion channels, Piezo1 and Piezo2 are hot topics of mechanosensitive molecules indispensable in skeletal tissue integrity and osteoporosis development. Limb osteoblast-specific Piezo1 knockout mice show less bone mineral density and trabecular network together with multiple fractures as compared to wild-type mice. Piezo1 loss induces osteoclastic erosion rather than bone formation. This molecule also “senses” tail suspension-induced bone deterioration. Furthermore, tamoxifen-mediated conditional knockout of Piezo1 in osteoblasts accelerates bone loss and osteoclastic erosion [65]. 

Piezo1 loss correlates with decreased osteogenic gene expression in human osteoporotic bone specimens. The inactivation of Piezo1 by RNA interference or Piezo family inhibitor GsMTX4 represses mechanically provoked cation current in osteoblastic cells. Mice deficient in Piezo1 driven by osteocalcin promoter show shorter leg bones and skull defect phenotypes together with less bone mineral density, trabecular structure, cortical thickness, and bone formation than wild-type mice. Likewise, Piezo1 knockout compromises mechanical loading-induced osteoblastic activity [66]. 

In osteocytes, Piezo1 loss inhibits flow fluid-mediated Ca^2+^ influx and osteocytic activity. Osteocyte-specific Piezo1 knockout mice also develop spontaneous fracture and poor bone quality in long bone compartments, including less bone mass, trabecular volume, strength, and mineral acquisition, etc. Skeletal tissue in these mice have a lower response to mechanical loading-induced bone mineral acquisition as compared to wild-type mice. Wnt, Yes1 associated transcriptional regulator (YAP), and Ca^2+^ involve Piezo1 control of osteocytic activities [67]. Piezo1 or Piezo1/2 double loss in mesenchymal progenitor cells inhibits cartilage, bone tissue development and bone formation rate. Piezo1/2 double knockout does not change the trabecular bone loss in botulinum toxin A induces skeletal muscle dystrophy as an in vivo model of unloading-induced osteoporosis [68].

In addition, transient receptor potential vanilloid (TRPV) signaling cascades involve mechanical stimulation-mediated bone homeostasis. TRPV4 disruption by RNA interference inhibits Ca^2+^ influx, repressing osteoclastogenic gene expression and osteoclast formation. Forced TRPV4 expression promotes osteoclastic activity. In vivo, lentivirus TRPV4 RNA interference mitigates estrogen deficiency-induced loss in bone mass, trabecular volume, thickness, and osteoclast number in skeletal tissue [69]. Microgravity downregulates intracellular Ca^2+^ influx and voltage sensitive Ca^2+^ channel signaling, inhibiting the metabolic activity of primary mouse osteoblasts [70].

### 4.2. Myokine FNDC5/Irisin

Fibronectin type III domain containing 5 (FNDC5) consists of an intracellular subunit and an extracellular irisin subunit. Physical exercise accelerates the cleavage of the extracellular subunit, which circulates around the peripheral blood. While FNDC5/irisin is largely produced in skeletal muscle, increasing evidence has revealed FNDC5/irisin and receptor αV integrin expression in bone-forming cells. FNDC5 knockout prevents estrogen deficiency-induced osteocyte loss and bone mass loss in mice [71]. Mechanical unloading by sciatic neurectomy or tail suspension inhibits FNDC5 expression in skeletal muscle and decreases the bone mineral density of tibiae. Flow shear stimulation promotes FNDC5 expression in C2C12 myoblasts [72].

Mice have high FNDC5 expression in bone tissue upon wheel running exercise (5000 m/day) for 2 consecutive weeks. Upregulated serum irisin is present in adiponectin knockout mice upon exercise. Administration with irisin recombinant protein promotes cortical bone thickness and osteogenic marker expression, including osterix, bone sialoprotein, and alkaline phosphatase in bone tissue [73]. 

Swimming exercises mitigates high-fat diet-mediated loss in bone mineral density and network of tibiae and femurs and serum irisin levels in rats. FNDC5 expression is also preserved in ovariectomized rat bone upon exercise for 6 weeks [74]. Microgravity inhibits osteogenic transcription factor expression, including osterix and Runx2, in osteoblasts. Irisin attenuates the deleterious effects of microgravity on bone-forming cells and increases osteoprotegrin, an osteoclast inhibitory cytokine [75].

### 4.3. Cytoskeleton

Cytoskeleton proteins, including actin, filament, and microtubules, etc., are well established molecules that are responsive to mechanical stimulation, inducing contractile and stiffness of cellular microcompartment. Microtubule actin crosslinking factor 1 (MACF1), a regulator for cytoskeleton dynamics, is required for bone tissue integrity. Conditional MACF1 knockout mice in osteoprogenitor cells show poor long bone and calvaria bone quality, including less bone mineral content, a lower bone formation rate, less bone mineral density and trabecular volume, and low osteogenesis of bone-marrow mesenchymal cells, compared to wild-type mice [76]. 

Mechanical unloading by hindlimb suspension represses the bone mineral density of tibiae and MACF1 expression in bone specimens. These effects are controlled by microRNA-138-5p as this microRNA disrupts MACF1 mRNA transcription and protein translation through targeting 3′-untranslated regions of MACF1. The preservation of MACF1 signaling by microRNA-138-5p antisense oligonucleotides compromises hindlimb suspension-induced bone loss [77]. 

Mechanical loading causes MC3T3-E1 osteoblasts and murine femurs to lose MACF1, one of cytoskeletal components. Loss of MCACF1 function by RNA interference inhibits osteoblast growth through downregulating the Wnt pathway component β-catenin signaling [78]. In vitro, the elasticity of the cytoplasmic microcompartment is changed in MC3T3-E1 osteoblasts incubated in a 10–30 g hypergravity condition. The cytoskeleton protein actin, rather than microtubules, is involved in these effects upon mechanical stress [79].

### 4.4. Focal Adhesion Kinase and Integrin

Focal adhesion kinase (FAK) and integrin also involve the bone tissue metabolism and remodeling under mechanical stimulation. These effects appear to depend on the bone-forming cell types. The deletion of FAK specifically in Dermo-1 expressing cells, a marker for dermal differentiation, inhibits the bone mineral density together with less osteoblast distribution than with wide-type mice. However, bone mass or osteoblast growth is unaffected in osteogenic precursor cells or osteoblast-specific FAK knockout mice [80].

FAK also mediates the histone deacetylase 4 (HDAC4)- or HDAC5-controlled dendritic process and sclerostin (a Wnt inhibitor) loss of osteocytes upon fluid flow shear stress as HDAC4 or HDAC5 knockout abolishes mechanical loading-mediated bone formation and β-catenin signaling. Inhibition of FAK by a specific inhibitor Defactinib (VS-6063) compromises the mechanical loading (cantilever bending loading)-induced promotion of osteocytic activities, including growth and extracellular matrix production, as well as reverses sclerostin secretion [81].

Pinch proteins regulate cytoskeleton assembly and cell mobility, as well as senses extracellular mechanical stimulation or stress. Osteoblast or osteocyte-specific Pinch1 or Pinch2 or Pinch1/2 double knockout mice have less bone mineral density, cortical bone thickness, trabecular bone volume, bone formation activity, and osteoblastogenesis of bone-marrow mesenchymal stem cells rather than osteoclast formation as compared to wild-type mice. The Pinch1 or Pinch2 deletion-mediated bone tissue deterioration is exacerbated in older animals. The loss in trabecular microstructure, cortical thickness, and bone mineral accumulation become worse in these knockout mice upon hindlimb suspension for 3 weeks [82].

Shuaib et al. establishes a mechano-agent base model revealing the link of heterogeneity of extracellular matrix integrin in bone cells in the presence of various mechanical stimulations [82]. Oscillatory fluid flow promotes αvβ3 integrin expression but increases osteoclastogenic cytokine RANKL expression in osteocytes. Estrogen withdrawal represses the promoting effects of mechanical stimulation on the integrin expression in osteocytes. A blockade of αvβ3 integrin by IntegriSense 750 compromises mechanical stimulation-mediated osteocytic activity [83].

Axial force, like loading, hypergravity, and microgravity, etc. is a widely studied model to characterize the contribution of mechanosensitive molecules to bone-forming cell behavior and function, bone tissue integrity, and osteoporosis development. Little is understood about the effects of oscillatory, pulsed, or electric stimulations, including vibration, ultrasound, and electromagnetic fields, etc. on the biological functions of these mechanosensitive molecules in bone-forming cells. In vitro or in vivo simulated models have revealed that a plethora of extracellular matrix or cytoskeleton or membrane proteins sense mechanical stimulation. The contribution of these molecules to cellular bioenergetics warrants characterization.

## 5. The Effects of Biophysical Stimulation on Mitochondrial Function

**Figure 2:**
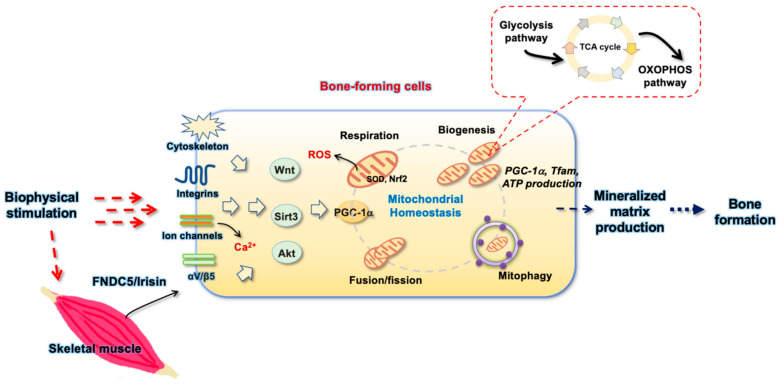
Schematic diagram showing the biophysical stimulation-induced modulation of mechanosensitive molecules-mediated mitochondrial homeostasis involved in bone forming cell anabolism and bone formation.

High throughput analytic approaches for characterizing gene transcription, protein footprints, and metabolic profiles prompt us to understand that multiple subcellular activities and signaling pathways are simultaneously changed in bone-forming cells to adapt mechanical stimulations or stresses. Sustained mitochondrial activity is important to meet the energy requirement for cellular anabolism upon mechanical modulation (Figure 2).

### 5.1. Transcriptome of Mitochondrial Metabolism in Mechanically Stressed Bone Cells

RNA-sequencing analysis and bioinformatics engines, including the Kyoto Encyclopedia of Genes and Genomes and Gene Ontology, etc., reveal that transcriptomic landscapes with thousands of gene transcription are simultaneously changed in mouse osteocytes incubated in microgravity conditions as an in vitro model of mechanical unloading. These genes involve mitochondrial glycolysis pathways, cell growth, and mechanotransduction pathways, etc., suggesting that microgravity affects mitochondrial glucose metabolism and respiratory activities [84]. 

The transcriptomic landmarks of bone-marrow mesenchymal stem cells incubated in simulated microgravity device (rotation speed, 0.1–10 rpm) depends on the extent of microgravity. Hundreds of gene transcription related to cytoskeletal protein bindings, tubule protein binding, and cell adhesion molecule binding are affected upon microgravity. Arginine, proline, tyrosine glucose, and vitamin metabolism are also changed in cell cultures [85]. Mechanical loading affects gene enrichment related to TCA cycle, cell–cell adhesion, and cell cycle together with Wnt, mitogen-activated protein kinase, and TGF-β1 pathways, etc., in murine tibiae tissue. The changes of these gene enrichment depend on the animals’ age [86].

### 5.2. Metabolome and Proteome of Mitochondrial Metabolism in Mechanically Stressed Bone Cells

Advanced liquid chromatography and tandem mass spectrometry show that mechanical stress changes metabolomic landmarks of osteocytes. Laminar fluid flow stress affects > 800 metabolites in cultured media of osteocytes. Of metabolites, cytidine, uridine, citrate, glycine, guanosine, inosine, etc., and ATP production are changed upon fluid flow stress [87]. Microgravity inhibits mitochondrial activity in osteocytes and alters 137 metabolites related to the TCA cycle, glycolysis, and malate-aspartate metabolism together with decreased glycerate phosphate and acyl-carnitine, as well as inhibits antioxidant GSSH levels. In the TCA cycle, acetyl-CoA and fumarate production is inhibited upon microgravity [88].

### 5.3. Mitochondrial Activity in Biophysical Stimulated Bone-Forming Cells

Microarray investigations reveal that mechanical loading promotes osteocytic gene expression of in situ osteocytes harvested from murine intracortical bone using laser capture microdissection approach. This biophysical manipulation also changes hundreds of gene transcription related to various metabolic activities [89]. 

The deletion of mitofusin 2, a mitochondrial membrane protein, specifically in osteocytes promotes cortical thickness; however, bone phenotypes are unaffected in these knockout mice upon mechanical loading. Mitochondrial morphology and respiration together with osteogenic differentiation are promoted in osteogenic progenitor cells from mitofusin 2 knockout mice [90]. Ca^2+^ transient is changed in MC3T3-E1 osteoblasts upon various magnitudes of fluid shear stress. Proteomic landscapes shoq that fluid shear stress activated the TCA cycle to promote mitochondrial energy production in osteoblasts [91].

### 5.4. Mitochondrial ROS and Oxidative Stress in Biophysical Modulation of Bone Cells

Mice overexpressing mitochondrial catalase show less lipid oxidation upon hindlimb unloading by tail suspension than wild type mice. Catalase overexpression fails to mitigate hindlimb unloading-induced cortical bone loss, suggesting that mitochondrial catalase-mediated redox is dispensable in “sensing” mechanical disuse in bone microenvironment [92]. Low-intensity pulsed ultrasound increases the ROS levels and osteogenic gene expression in MC3T3-E1 osteoblasts. The inhibition of ROS by diphenylene iodonium attenuates these effects [93]. In mesenchymal stem cells, 30–90 nm, 1000 Hz nanovibration promotes osteogenic gene expression and osteoblastogenesis. This biophysical stimulation increases Piezo2 and TRPV1 expression, as well as upregulates the mitochondrial metabolism, including glycolysis and the TCA cycle and ROS generation [94].

## 6. Treatment Options for Osteoporosis via Mitochondrial Energetics and ROS

The anabolic effects of mechanosensitive molecules, mitochondrial metabolism regulators and antioxidants on bone-forming cells upon biophysical stimulation indicate that modulation of these molecules may have remedial potential for osteoporosis development. Control of transcriptional coactivator peroxisome proliferator-activated receptor gamma coactivator-1α (PGC-1α), a key player of mitochondrial biogenesis, influences the development of osteoporosis. 

Physical exercise promotes PGC-1α expression, whereas hindlimb unloading induces skeletal muscle atrophy together with low PGC-α loss worsens the extent of estrogen deficiency-induced osteoporosis [95]. Transgenic overexpression of this molecule reverses bone loss in ovariectomized mice [96]. Agonists for the sirtuin family, irisin recombinant protein, and Nrf2 activator are found to prevent skeletal tissue from mechanical disuse or unloading-induced microarchitecture deterioration.

### 6.1. The Sirtuin Family Control of Bone Integrity

Activation of PGC-1α by the sirtuin pathway appears to have protective effects on bone tissue. The sirtuin family is found to promote the osteogenic potential of mesenchymal cells or osteoblastic activity by enhancing PGC-1α, FOXO3, SOD2, and oxidative phosphorylation [97]. Mice overexpressing Sirt1 specifically in mesenchymal stem cells develop more skeletal size, bone mass, bone formation rate, and osteogenic differentiation potential than wild type mice. Sirt1 overexpression promotes FOXO3 signaling and SOD2 levels in osteogenic progenitor cells [98].

Osteoblast-specific Sirt6 knockout mice have phenotypes of low bone mass together with increased osteoclast resorption. Sirt6 loss affects osteoclastogenic rather than osteoblast differentiation capacity in the bone microenvironment [99]. A study of Li et al., shows that Sirt3 loss interrupts mitochondrial activity and osteoclast formation. Sirt3 knockout mice upon aging or ovariectomy show high bone mass as compared to wild-type mice [100]. A randomized placebo-controlled trial reveal that menopausal women upon 12-month resveratrol, a Sirt3 agonist derived from plants, supplementation have better bone mineral density of the lumbar spine and femoral neck together with less serum bone resorption maker than the placebo group [101].

Administration with resveratrol for 2 months compromises age-induced loss in bone mass, mineral acquisition, and bone formation in SAMP6 mice as an in vivo model of senile osteoporosis. This agent improves the mitochondrial ATP production, membrane potential, ROS generation, and osteogenesis of bone-marrow mesenchymal cells from SAMP6 mice through regulating mitochondrial inner membrane integrity [102]. Administration with resveratrol also attenuates glucocorticoid excess-induced oxidative stress and apoptosis of bone-marrow stromal cells through promoting PGC-1α signaling, as well as prevents rats from developing femoral head microstructure damage upon high doses of dexamethasone treatment [103].

### 6.2. Irisin Recombinant Protein Promotion of Bone Mass

Accumulating evidence has shown that myokine irisin signaling, a soluble fragment hydrolyzed from FNDC5, involves PGC-1α signaling, which promotes mitochondrial biogenesis and metabolism to delay tissue deterioration [104]. Aerobic exercise also promotes FNDC5/irisin and PGC-1α signaling, slowing down the development of kidney and cardiac injury. Irisin recombinant protein attenuates hydrogen peroxide (H_2_O_2)_-induced oxidative stress and cell dysfunction [105]. 

Likewise, osteogenic cell-specific Fndc5 knockout mice have phenotypes of low bone mineral density, decreased serum irisin levels, and less osteogenic differentiation of bone-marrow mesenchymal cells than the wild-type mice. Fndc5 loss compromises the promoting effect of exercise on bone mass. Irisin recombinant protein and exercises preserve the loss in bone mineral density and strength in Fndc5 knockout mice [106]. Upon downhill running exercise for 8 weeks, ovariectomized mice have more trabecular bone mineral density and osteoblast number together with increased serum irisin levels and FNDC5 expression in bone tissue as compared to control ovariectomized animals. Administration with irisin receptor agonist cyclo RGDyk protein strengthens the protective effects of exercise on bone tissue integrity in ovariectomized mice [107].

Irisin recombinant protein treatment delays hindlimb suspension-induced cortical and trabecular bone loss and skeletal muscle atrophy in mice [108]. This recombinant protein also represses inflammatory cytokine-induced loss in mitochondrial activity, including ATP production, mitophagy, and ROS production, as well as improves subchondral bone loss in osteoarthritic knees [109].

## 7. Conclusions

This article offers new molecular insight into how bone-forming cells transduce biophysical stimulations into intracellular signaling, which modulates the mitochondrial metabolism and ROS homeostasis for bone anabolism. Randomized controlled trials have shown the positive effects of physical exercise or whole-body vibration on preventing skeletal tissue deterioration. While electromechanical stimulation interventions improve bone loss in experimental osteoporosis models, whether these investigations can be extrapolated to human osteoporosis and whether local biophysical stimulation have ubiquitous effects on whole-body bone tissue remains uncertain. 

Emerging evidence of bone-forming cell-specific knockout mice and transgenic mice have uncovered the biological roles of a plethora of new mechanosensitive molecules in mediating mechanical stimuli/stresses, which influences the bone microarchitecture integrity and osteoporosis development. Proof-of-concept studies reveal that the promotion of the mitochondrial metabolism by sirtuin agonists or irisin recombinant protein has remedial effects to estrogen deficiency-induced osteoporosis or biophysical disuse-mediated bone deterioration. This review highlights the conversion of mechanical stimulation into bioenergetics, which has remedial potential for preventing bone-forming cell dysfunction and osteoporotic disorders.

## Figures and Tables

**Table 1:** Effects of biophysical stimulation on osteoporosis development in human and in laboratory animals.

Types	Participants	Protocols	Response of Skeletal Tissue	References
Physical activity; regular exercise	Senior people; postmenopausal women; middle-aged males, etc.	Walking, stepping, lower extremity muscle strength; high-intensity resistance and impact training; team handball practices; dynamic resistance exercise	Improvement of mobility, gait and muscle strength; improvement of spinal, hip and distal radius bone mineral density and reduction of osteoporotic fracture risk	[32,33,34,35,36,37]
Electromechanical vibration	Postmenopausal women; artistic swimmers,	Whole-body vibration	Improvement of tibial, femoral neck, lumbar bone mineral density and stiffness	[38,39,40,41]
	Aged rats, diabetic rabbits	Low magnitude whole body vibration	Improvement of trabecular and cortical bone mineral density, microstructure, and strength	[42,43,44]
Mechanical loading	Healthy women; postmenopausal women	Voluntary upper extremity compressive loading; one-legged jumping	Improvement of ultradistal radius bone mineral density; bone material strength index	[45,46]
	Ovariectomized mice; young or aged mice	Knee loading; axial loading; spinal loading	Improvement of tibial bone mineral density, microstructure,	[47,48,49]
Piezoelectric stimulation	Ovariectomized mice or rats; rats with spinal cord injury	Pulsed electromagnetic fields, low-intensity ultrasound	Improvement of trabecular and cortical bone loss, strength,	[50,51,52]

**Table 2:** Bioinformatics Cytoscape (http://cytoscaple.org (accessed on 27 July 2021)) and Genecards (www.genecards.org (accessed on 27 July 2021)) predication of mechanosensitive molecules involving gene-gene interaction and intracellular pathways.

Types	Name	Gene-Gene Interaction	Signaling Pathways
Ion channel	*PIZO1*	*GCM1, ESSRA, SMAD4, PU1, SPI1*, etc.	IL-6 receptor family pathway, TGF-β receptor family pathway, TNF receptor pathway, androgen receptor, estrogen receptor, glucocorticoid receptor, peroxisome proliferator-activated receptors, retinoic acid receptors, retinoid X receptors, vitamin D receptor, checkpoint kinases, cyclin-dependent kinases, methyltransferases, Zinc finger-like transcription factor, and FOXA family.
Ion channel	*TRPV4*	*PRKACA, SGK1, LYN*,	Collagen receptor family, fibroblast growth factor receptors, hepatocyte growth factor receptors, interferon receptor family, PDGF receptor, RET family, toll-like receptors, VEGF receptors, androgen receptor, estrogen receptors, Liver X receptors, peroxisome proliferator-activated receptors, retinoic acid receptors, nuclear receptor coactivator, histone deacetylase, Ab1 family kinases, cyclin-dependent kinases, RAF kinases, ribonucleoside-diphosphate reductases, and CREB-like factors.
Myokine	*FNDC5*/Irisin	A*TP2A3, SLC9A1, RHBDD3, GHITM, EDOD1, TMCO3, GNAL, NHLH1, SF1, TFAP4, UBP1, HEB, LBP, ESSRA, E2A, HEN1, TCF12, TCF3, REPIN1, PAX4, MyoD, AP*4, etc.	HGF receptor pathway; TGF-β receptor pathway; TNF receptor pathway, retinoic acid receptor pathway, vitamin D receptor pathway, cyclin-dependent kinases, FOXA family, etc.
Cytoskeleton	*MACF*1	*TCF3, SREB, TCF4, RXRA, FXR, E47, NR1H4, HEB, ZIC, PXR, E2A, TAL1, TCF12*, etc.	Collagen receptor family, epidermal growth factor receptors, IL-6 receptor family, TNF receptors, 5-hydroxytryptamine receptors, androgen receptor, estrogen receptors, glucocorticoid receptor, retinoid acid receptors, Ab1 family kinases, cyclin-dependent kinases, nucleotidyltransferases, FOXA family, TCF7, nuclear receptor compressor, and β-catenin.
